# Analysis of risk factors and prognostic factors of brain metastasis in gastric cancer: a surveillance, epidemiology and end-results database study

**DOI:** 10.1038/s41598-023-46109-9

**Published:** 2023-10-31

**Authors:** Mohammad Ebad Ur Rehman, Afifa Kulsoom, Fatima Faraz, Biah Mustafa, Abia Shahid, Huzaifa Ahmad Cheema, Shahzaib Maqbool, Israr Khan, Taimoor Hussain, Ahmed Iftikhar, Rehmat Ullah Awan, Sarya Swed, Shahzad Raza, Faiz Anwer

**Affiliations:** 1 Department of Medicine, Rawalpindi Medical University, Rawalpindi, Pakistan; 2 Department of Community Medicine, Rawalpindi Medical University, Rawalpindi, Pakistan; 3 Department of Medicine, King Edward Medical University, Lahore, Pakistan; 4 Department of Medicine, HMH Palisades Medical Center, North Bergen, NJ USA; 5 Bolan Medical Complex Hospital, Quetta, Pakistan; 6 Department of Medicine, The University of Arizona, Tucson, AZ USA; 7 Department of Medicine, Ochsner Rush Medical Center, Meridian, MS USA; 8 Faculty of Medicine, Aleppo University, Aleppo, Syria; 9 Department of Hematology and Medical Oncology, Taussig Cancer Institute, Cleveland Clinic, Cleveland, OH USA

**Keywords:** Cancer, Diseases, Gastroenterology, Oncology, Risk factors

## Abstract

Brain metastasis in gastric cancer (GC) patients is a rare phenomenon that is associated with adverse clinical outcomes and poor survival rates. We conducted a retrospective cohort study to investigate the incidence, risk factors and prognostic factors of brain metastasis in GC patients. Data on sociodemographic and tumor characteristics of GC patients from 2010 to 2019 was extracted from the Surveillance, Epidemiology and End-Results (SEER) database. Descriptive statistics, multivariable logistic and Cox regression were applied on SPSS. Kaplan–Meier-Survival curves and ROC curves were constructed. A total of 59,231 GC patients, aged 66.65 ± 13.410 years were included. Brain metastasis was reported in 368 (0.62%) patients. On logistic regression, the risk of brain metastasis was significantly greater in males, patients aged < 60 years and patients having concurrent bone and lung metastasis. High grade and high N stage were significant risk factors for development of brain metastasis. Patients who had undergone surgery for the primary tumor were at reduced risk for brain metastasis (adjusted odds ratio 0.210, 95% CI 0.131–0.337). The median OS was 3 months in patients with brain metastasis and 17 months in patients without brain metastasis (p < 0.05). On Cox regression, Grade IV tumors and primary antral tumors were significant predictable parameters for poor prognosis. Overall Survival (OS) and Cancer-Specific Survival (CSS) were prolonged in patients who had undergone surgery. Brain metastasis in gastric cancer is associated with significantly worse survival. Employing large-scale screening for high-risk patients holds a promising impact to improve survival rates, but it must be accurately balanced with a comprehensive understanding of clinicopathological aspects for accurate diagnosis and treatment.

## Introduction

The World Health Organization ranks stomach cancer as the fifth most common form of cancer worldwide. According to the latest statistics by GLOBOCAN2020, gastric cancer remains the fourth-leading cause of death worldwide, accounting for 7.7% of cancer deaths, and is a significant contributor to global mortality and morbidity, particularly in men^1^. Being extremely aggressive and heterogeneous, gastric cancer poses a global public health risk^2^. It is a multifactorial disease brought on by a variety of genetic and environmental factors. Owing to the vague symptoms and lack of proper awareness and understanding of the disease, the cancer usually has a low survival rate. It is commonly detected after it has spread to one or more organs^3^. Despite rapid advancements in diagnostic and therapeutic options, fewer than 5% of patients survive more than 5 years after initial diagnosis^4,5^.

The brain is a common site of metastatic spread in several malignancies. According to Lemke et al., nearly 10% of all cancer patients develop brain metastasis at some point in the course of the disease^6,7^. This, however, is mostly dependent on the location of the first tumor, as a majority of brain metastases only involve the tumors of skin, breast, and lungs. As much as 4% of esophageal and colorectal cancers also metastasize to the brain. On the other hand, with an incidence of less than 1% (0.2–0.7%), the brain is a very uncommon site for metastasis in gastric cancer^6^. Gastric cancers spread more frequently to the liver (48%), peritoneum (32%), bones (12%) and lungs (15%)^8,9^.

Although brain metastasis is rare in cases of gastric cancer, it has an unfavourable prognosis globally and low survival rates. Patients are severely cachexic, and the median Overall Survival (OS) is reported to be 1.3–2.4 months, due to its detection at an advanced stage of the disease^10–12^. After a diagnosis of gastric cancer, the median time until brain metastasis is reported to be 12.3 months^10^. Most of these patients have their cancer already metastasized to bones, liver and lungs^12^. Although resecting the metastatic lesion significantly increases the patients' chances of survival, brain metastasis is usually very difficult to treat and resect^10,11^. Lin et al. identified size, tumor extension, T, and N stages as significant risk factors for metastatic spread to the brain^13^. Analysis of the Surveillance, Epidemiology and End-Results (SEER) registry from 1998 to 2004 revealed that age, sex, and the location of the primary stomach tumor all significantly predicted the prognosis of patients with metastatic gastric cancer^14^.

Despite the poor prognosis in patients who develop brain metastasis, there is limited literature which describes the risk factors for brain metastasis and the prognostic factors which influence survival. The available evidence consists primarily of reviews, case series and small-scale observational studies. Therefore, we undertook an analysis of data from the SEER database to correctly identify the risk factors as well as prognostic factors for brain metastasis in gastric carcinoma.

## Methods

### Data source

We extracted data for our retrospective cohort study from the SEER database, which is a data registry funded by the National Cancer Institute. We used the dataset Incidence-SEER 17 Regs Research Data, Nov 2021 Sub (2000–2019) to generate case listings^14^. Data on the metastatic spread to distant organs, including the brain, became available from 2010. Therefore, we used SEER*Stat 8.4.0.1 software to identify the pathologically diagnosed cases of gastric cancer from 2010 to 2019^15^. We selected patients based on the following inclusion criteria: (1) diagnosis of gastric cancer (site recode ICD-O-3/WHO2008:C160-C169), (2) complete metastatic and survival information. Patients with cancer diagnosed during autopsy or through their death certificate and those with incomplete survival data were excluded.

### Outcome variables

We collected information pertaining to gender, race, age at diagnosis, year of diagnosis, primary site, histology, tumor size, grade, TNM stage, distant organ metastasis, surgery of primary tumor, income, OS and Cancer-Specific Survival (CSS). The time interval between diagnosis of gastric cancer and death due to any cause was defined as OS. The time interval between diagnosis of gastric cancer and death due to gastric cancer was defined as CSS. Authorization was obtained from the SEER website and no additional ethical approval was required.

### Statistical analysis

The sociodemographic and tumor characteristics of included patients were summarized using descriptive statistics. Comparisons were made between patients with and without brain metastasis using the chi-square test. Risk factors for brain metastasis in gastric cancer patients were discerned using logistic regression analysis. Receiver Operator Characteristics (ROC) curves were constructed and Areas Under the Curve (AUC) were computed to determine the diagnostic efficacy of multiple variables in predicting brain metastasis. Survival function estimation and comparison among patients with and without brain metastasis were performed using Kaplan–Meier estimates and the log-rank test. Variables that were associated with prognosis in gastric cancer patients with brain metastasis were identified using Cox proportional hazard regression. All statistical analysis was performed on SPSS version 26, and a p-value less than 0.05 was considered statistically significant.

## Results

### General data

A total of 59,231 patients with gastric cancer were enrolled in this study, including 35,889 (60.6%) males and 23,342 (39%) females. 70.9% of patients (n = 41,995) were Caucasians, 13.1% (n = 7776) were African American and 16% (9460) from other ethnicities. Metastasis to the brain was reported in 368 (0.62%) patients.

### Characteristics of patients with or without metastasis

It was found that brain metastasis was significantly more common in patients aged younger than 60 years, males, Caucasians, patients with primary tumor site of the cardia, grade IV, unknown T stage, unknown N stage, patients with no previous surgery for primary tumor and in whom cancer had metastasized to other organs such as bone, liver and lung (*P* < 0.05). There was no significant association between income and histological type of tumor and brain metastasis (Table 1).

**Table 1:** Clinicopathological factors of patients with and without brain metastasis.

Characteristics	No brain metastasis N = 58,863	Brain metastasis N = 368	*X*^*2*^	P value
Age			17.222	< 0.001
≤ 60 years	17,949 (99.2%)	149 (0.8%)		
> 60 years	40,914 (99.5%)	219 (0.5%)		
Sex			20.222	< 0.001
Male	35,624 (99.3%)	265 (0.7%)		
Female	23,239 (99.6%)	103 (0.4%)		
Race			18.324	< 0.001
Caucasian	41,697 (99.3%)	298 (0.7%)		
African American	7746 (99.6%)	30 (0.4%)		
Others	9420 (99.6%)	40 (0.4%)		
Tumor site			101.328	< 0.001
Cardia	17,400 (99.0%)	182 (1.0%)		
Antrum	9642(99.8%)	16 (0.2%)		
Body	6649 (99.6%)	24 (0.4%)		
Fundus	3066 (99.4%)	19 (0.6%)		
Greater	2635 (99.6%)	11 (0.4%)		
Lesser	4287 (99.6%)	16 (0.4%)		
Pylorus	1364 (99.9%)	2 (0.1%)		
Not specified	13,820 (99.3%)	98 (0.7%)		
Histological type			6.884	0.142
Adenocarcinoma	41,251 (99.4%)	267 (0.6%)		
Mucinous	794 (99.7%)	2 (0.3%)		
Papillary	187 (98.9%)	2 (1.1%)		
Signet ring cell	8938 (99.3%)	62 (0.7%)		
Other	7693 (99.5%)	35 (0.5%)		
Grade			35.421	< 0.001
Grade I	4885 (99.94%)	3 (0.06%)		
Grade II	11,760 (99.4%)	69 (0.6%)		
Grade III	26,940 (99.4%)	169 (0.6%)		
Grade IV	914 (99.1%)	8 (0.9%)		
Unknown	14,364 (99.2%)	119 (0.8%)		
Tumor size			115.675	< 0.001
≤ 5 cm	23,475 (99.7%)	80 (0.3%)		
> 5 cm	12,649 (99.6%)	45 (0.4%)		
Others	22,739 (98.9%)	243 (1.1%)		
T stage			226.769	< 0.001
T1	14,042 (99.6%)	53 (0.4%)		
T2	6545 (99.8%)	12 (0.2%)		
T3	13,203 (99.7%)	39 (0.3%)		
T4	9209 (99.6%)	37 (0.4%)		
Unknown	15,864 (98.6%)	227 (1.4%)		
N stage			124.920	< 0.001
No	30,633 (99.6%)	118 (0.4%)		
N1	11,769 (99.2%)	100 (0.8%)		
N2	4179 (99.7%)	11 (0.3%)		
N3	3998 (99.4%)	23 (0.6%)		
Unknown	8284 (98.6%)	116 (1.4%)		
Surgery			244.251	< 0.001
No	31,351 (98.1%)	346 (1.1%)		
Yes	27,512 (99.92%)	22 (0.08%)		
Bone metastasis			720.662	< 0.001
No	56,509 (99.6%)	250 (0.4%)		
Yes	2353 (95.2%)	118 (4.8%)		
Liver metastasis			184.186	< 0.001
No	50,302 (99.6%)	222 (0.4%)		
Yes	8561 (98.3%)	146 (1.7%)		
Lung metastasis			757.173	< 0.001
No	56,156 (99.6%)	238 (0.4%)		
Yes	2707 (95.4%)	130 (4.6%)		
Income			2.131	0.345
Less than 50 k	7872 (99.4%)	44 (0.6%)		
50–75 k	32,012 (99.3%)	214 (0.7%)		
More than 75 k	18,979 (99.4%)	110 (0.6%)		

### Univariable and multivariable logistic regression analysis of odds of gastric cancer brain metastasis

Univariable analysis results suggested that tumor site in stomach, race, age, gender, T stage, N stage, tumor grade, metastasis to bone, liver and lung, surgery of primary tumor, tumor size and histological type were significant independent risk factors for brain metastasis (*P* < 0.05), whereas income was not a significant risk factor for brain metastasis (*P* > 0.05) (Table 2).

**Table 2:** Univariable and multivariable logistic regression analysis for risk factors of brain metastasis in patients with gastric cancer.

Parameter	Univariable analysis		Multivariable analysis	
	COR	95% CI	AOR	95% CI
Age				
Less than 60 years	1 [reference]	–	1 [reference]	–
Greater than 60 years	0.645#	0.523–0.795	0.680#	0.547–0.846
Gender				
Female	1 [reference]	–	1 [reference]	–
Male	1.678#	1.336–2.109	1.294#	1.017–1.647
Race				
Caucasian	1 [reference]	–	1 [reference]	–
African American	0.542#	0.372–0.789	0.703	0.478–1.035
Other	0.594#	0.427–0.827	0.807	0.574–1.136
Bone	11.335#	9.072–14.164	3.845#	3.020–4.896
Liver	3.846#	3.132–4.768	1.139	0.898–1.446
Lung	11.331#	9.119–14.080	3.943#	3.101–5.013
Surgery	0.072#	0.047–0.112	0.210#	0.131–0.337
Grade				
Grade-I	1 [reference]	–	1 [reference]	–
Grade-II	9.554#	3.006–30.367	4.387#	1.367–14.081
Grade-III	10.215#	3.260–32.003	3.819#	1.204–12.109
Grade-IV	14.252#	3.774–53.823	7.961#	2.049–30.921
Unknown	13.490#	.228–42.444	5.420#	1.704–17.238
Histology				
Adenocarcinoma	1.423#	0.999–2.025	1.081	0.741–1.575
Mucinous	0.554	0.097–1.567	0.413	0.098–1.748
Papillary	2.351	0.408–6.691	3.361	0.751–15.042
Signet ring cell	1.525	0.812–1.414	1.194	0.764–1.864
Other	1 [reference]	–	1 [reference]	–
Site				
Cardia	1 [reference]	–	1 [reference]	–
Antrum	0.159#	0.095–0.265	0.267#	0.158–0.452
Body	0.345#	0.225–0.529	0.530#	0.340–0.827
Fundus	0.592#	0.369–0.952	0.888	0.545–1.446
Greater curvature	0.399#	0.217–0.734	0.741	0.395–1.389
Lesser curvature	0.357#	0.214–0.595	0.652	0.385–1.104
Pylorus	0.140#	0.035–0.565	0.281	0.069–1.145
Not specified	0.678#	0.530–0.869	0.653#	0.495–0.860
N				
No	1 [reference]	–	1 [reference]	–
N1	2.206#	1.689–2.882	1.239	0.930–1.651
N2	0.683	0.368–1.269	1.008	0.531–1.915
N3	1.493	0.954–2.337	2.527#	1.548–4.127
Unknown	3.635#	2.810–4.702	1.459#	1.110–1.917
Tumor size				
≤ 5 cm	1 [reference]	–	1 [reference]	–
> 5 cm	1.044	0.724–1.505	0.868	0.595–1.268
Others	3.136#	2.434–4.040	1.254	0.955–1.647
Income				
Less than 50 k	1 [reference]	–		
50–75 k	1.196	0.864–1.656		
> 75 k	1.037	0.730–1.472		
T stage				
T1	1 [reference]	–		
T2	0.486#	0.259–0.910	.674	.357–1.273
T3	0.783	0.517–1.184	.769	.498–1.189
T4	1.064	0.699–1.621	.920	.592–1.430
Others	3.791#	2.809–5.117	1.513#	1.099–2.083

^#^Indicates p value less than 0.05

All significant factors from the univariable analysis were entered in to the multivariable logistic regression model. Risk of brain metastasis was significantly lower in patients older than 60 years, compared to patients younger than 60 years (adjusted odds ratio [aOR] 0.680, 95% CI 0.547–0.846). Males were at a significantly higher risk of brain metastasis compared to females (aOR 1.294, 95% CI 1.017–1.647). Risk of brain metastasis was significantly greater in patients who had developed metastasis to lung (aOR 3.943, 95% CI 3.101–5.013) and bone (aOR 3.845, 95% CI 3.020–4.896). Patients with Grade II cancer (aOR 4.387, 95% CI 1.367–14.081), Grade III cancer (aOR 3.819, 95% CI 1.204–12.109), Grade IV cancer (aOR 7.961, 95% CI 2.049–30.921) and patients with unknown grade (aOR 5.420, 95% CI 1.704–17.238) were more likely to develop metastasis to brain as compared to patients with Grade I tumors. Patients who underwent surgery of primary tumor were at significantly lower risk of brain metastasis (aOR 0.210, 95% CI 0.131–0.337). Risk of brain metastasis was lesser in patients with primary tumor of antrum (aOR 0.267, 95% CI 0.158–0.452) and body (aOR 0.530, 95% CI 0.340–0.827), compared to cardia. Patients at N3 stage (aOR 2.527, 95% CI 1.548–4.127) and patients with unknown N stage (aOR 1.459, 95% CI 1.110–1.917) were at greater risk of brain metastasis, compared to patients at N_1_ stage. Patients who had undergone previous surgery for primary tumor were at lesser risk of developing brain metastasis as compared to patients who had not (aOR 0.210, 95% CI 0.131–0.337). Race, histology, liver metastasis, tumor size and T stage were not significant predictors of brain metastasis (Table 2).

### Comparison of diagnostic efficacy of risk factors for gastric cancer brain metastasis

**Figure 1:**
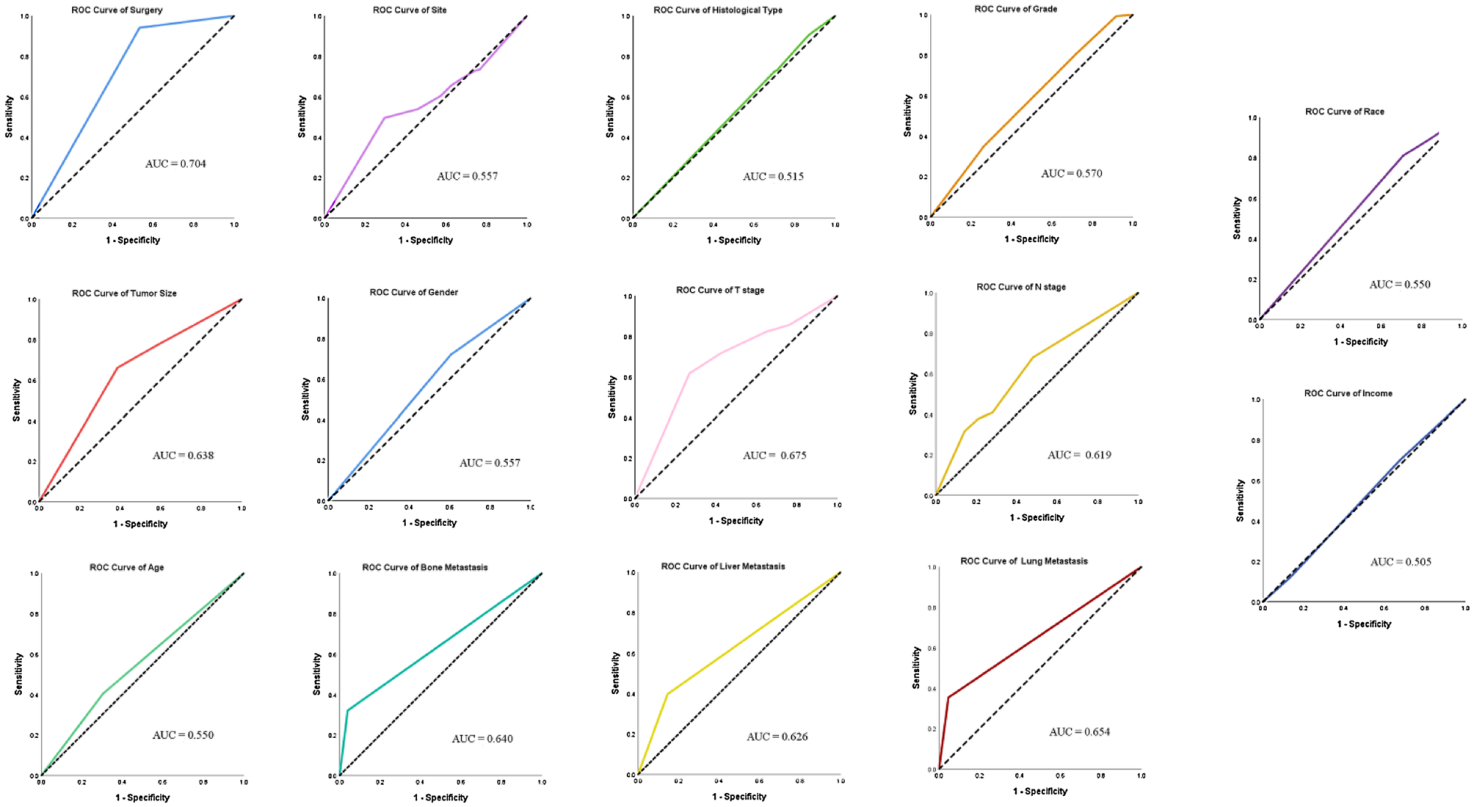
ROC curves for risk factors predicting brain metastasis in gastric cancer.

We constructed ROC curves to determine the diagnostic power of risk factors for brain metastasis in gastric cancer patients (Fig. 1). The AUC for different risk factors were compared. The results showed that the AUC for surgery was 0.704 (95% CI 0.684–0.724, *p* < 0.001), AUC for site was 0.557 (95% CI 0.523–0.590, *p* < 0.001); AUC for histological type of tumor was 0.515 (95% CI 0.487–0.544, *p* = 0.305); the AUC for grade was 0.570 (95% CI 0.542–0.598, *p* < 0.001); the AUC for tumor size was 0.638 (95% CI 0.610–0.666, *p* < 0.001); the AUC for gender was 0.557 (95% CI 0.529–0.586, *p* < 0.001); the AUC for T stage was 0.675 (95% CI 0.646–0.704, *p* < 0.001); the AUC for N stage was 0.619 (95% CI 0.590–0.649, *p* < 0.001); the AUC for age was 0.550 (95% CI 0.520–0.580, *p* = 0.001); the AUC for bone metastasis was 0.640 (95% CI 0.607–0.674, *p* < 0.001); the AUC for liver metastasis was 0.626 (95% CI 0.594–0.658, *p* < 0.001); the AUC for lung metastasis was 0.654 (95% CI 0.620–0.687, *p* < 0.001); the AUC for race was 0.550 (95% CI 0.522–0.578, *p* = 0.001); the AUC for income was 0.505 (95% CI 0.477–0.534, *p* = 0.721). Surgery of primary tumor was the best diagnostic predictor for brain metastasis.

### Survival analysis

**Figure 2:**
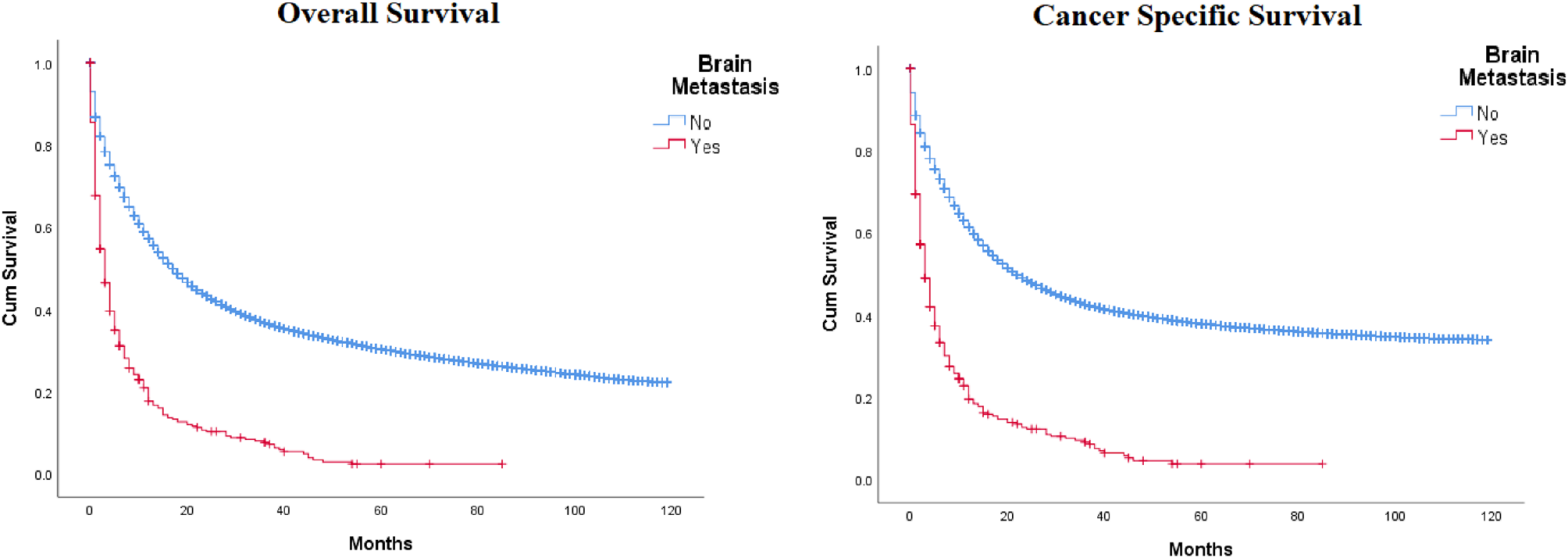
KM curves for OS and CSS in gastric cancer patients with and without brain metastasis.

The median OS was 3.0 months (95% CI 2.449 ~ 3.551) in patients with brain metastasis and 17 months (95% CI 26.778 ~ 33.222) in patients without brain metastasis (p < 0.05) (Fig. 2). The 1, 2, and 4-year OS of patients with brain metastasis were 14.4%, 8.42%, and 1.62%, respectively. The 1, 2 and 4-year OS of patients without brain metastasis were 49%, 33%, and 17.96%, respectively. The median CSS of patients with brain metastasis was 3.0 months (95% CI 2.313 ~ 3.687). For patients without brain metastasis, the median CSS was 22 months (95% CI 21.397 ~ 22.603) (p < 0.05).

### Univariable and multivariable analysis of factors affecting the prognosis of patients with brain metastasis in gastric cancer

Univariable Cox regression analysis of patients with brain metastasis showed that bone metastasis, no previous surgery, grade 4 tumor, unknown N stage and primary tumor of antrum, body, greater curvature and overlapping/non-specified site of primary tumor were significant predictors of worse OS (Tables 3, 4).

**Table 3:** AUROC analysis for diagnostic power of predictors of brain metastasis in gastric cancer patients

Parameter	AUC (95% CI)	P value
Surgery	0.704 (0.684–0.724)	< 0.001
Site	0.557 (0.523–0.590)	< 0.001
Histological type of tumor	0.515 (0.487–0.544)	0.305
Grade	0.570 (0.542–0.598)	< 0.001
Tumor size	0.638 (0.610–0.666)	< 0.001
Gender	0.557 (0.529–0.586)	< 0.001
T stage	0.675 (0.646–0.704)	< 0.001
N stage	0.619 (0.590–0.649)	< 0.001
Age	0.550 (0.520–0.580)	0.001
Bone metastasis	0.640 (0.607–0.674)	< 0.001
Liver metastasis	0.626 (0.594–0.658)	< 0.001
Lung metastasis	0.654 (0.620–0.687)	< 0.001
Race	0.550 (0.522–0.578)	0.001
Income	0.505 (0.477–0.534)	0.721

**Table 4:** Univariable and multivariable cox regression analysis to predict survival in gastric cancer patients with brain metastasis.

Parameter	Overall survival				Cancer specific survival			
	Univariable		Multivariable		Univariable		Multivariable	
	OR (95% CI)	P-value	OR (95% CI)	P-value	OR (95% CI)	P-value	OR (95% CI)	P-value
Age								
Less than 60 years	1 [ref]	–			1 [ref]	–		
Greater than 60 years	0.978 (0.786–1.218)	0.843			0.954 (0.761–1.196)	0.684		
Gender								
Female	1 [reference]	–			1 [reference]	–		
Male	0.929 (0.731–1.182)	0.550			0.975 (0.758–1.253)	0.842		
Race								
Caucasian	1 [reference]	–			1 [reference]	–		
African American	1.346 (0.897–2.020)	0.151			1.195 (0.771–1.852)	0.426		
Other	0.997 (0.703–1.413)	0.986			0.964 (0.670–1.386)	0.843		
Bone	1.363 (1.084–1.714)	0.008	1.255 (0.994–1.586)	0.057	1.404 (1.109–1.776)	0.005	1.304 (1.025–1.659)	0.031
Liver	1.108 (0.888–1.382)	0.364			1.082 (0.861–1.361)	0.499		
Lung	1.103 (0.881–1.383)	0.393			1.128 (0.894–1.423)	0.311		
Surgery	0.452 (0.273–0.748)	0.002	0.452 (0.269–0.758)	0.003	0.454 (0.270–0.765)	0.003	0.458 (0.268–0.781)	0.004
Grade								
Grade-I	1 [reference]	–	1 [reference]	–	1 [reference]	–	1 [reference]	–
Grade-II	2.143 (0.522–8.797)	0.290	2.274 (0.551–9.389)	0.256	1.970 (0.479–8.106)	0.340	2.071 (0.5–8.574)	0.315
Grade-III	3.925 (0.969–15.901)	0.055	3.452 (0.843–14.129)	0.085	3.728 (0.920–15.112)	0.657	3.324 (0.811–13.620)	0.095
Grade-IV	5.799 (1.224–27.470)	0.027	5.322 (1.114–25.415)	0.036	5.774 (1.219–27.358)	0.027	5.287 (1.106–25.268)	0.037
Unknown	3.661 (0.901–14.872)	0.070	2.869 (0.696–11.832)	0.145	3.352 (0.824–12.633)	0.091	2.717 (0.658–11.226)	0.167
Histology								
Other	1 [reference]	–			1 [reference]	–		
Adenocarcinoma	0.786 (0.537–1.150)	0.215			0.800 (0.540–1.186)	0.266		
Mucinous	0.789 (0.188 – 3.306)	0.746			0.846 (0.201–3.555)	0.819		
Papillary	0.189 (0.026–1.394)	0.102			0.215 (0.029–1.588)	0.132		
Signet ring cell	1.155 (0.743–1.794)	0.522			1.136 (0.719)–1.796)	0.584		
Site								
Cardia	1 [reference]	–	1 [reference]	–	1 [reference]	–	1 [reference]	–
Antrum	1.845 (1.084–3.139)	0.024	1.957 (1.122–3.414)	0.018	1.784 (1.029–3.091)	0.039	1.858 (1.045–3.302)	0.035
Body	1.760 (1.100–2.814)	0.018	1.477 (0.910–2.398)	0.114	1.724 (1.065–2.790)	0.027	1.431 (0.871–2.350)	0.157
Fundus	1.429 (0.852–2.399)	0.176	1.279 (0.752–2.174)	0.363	1.384 (0.811–3.262)	0.233	1.229 (0.712–2.214)	0.459
Greater	2.479 (1.337–4.596)	0.004	2.339 (1.238–4.422)	0.009	2.093 (1.602–4.124)	0.033	1.963 (0.978–3.9410	0.058
Lesser	1.375 (0.809–2.338)	0.240	1.142 (0.657–1.984)	0.638	1.356 (0.783–2.346)	0.277	1.102 90.623–1.950)	0.738
Pylorus	0.873 (0.216–3.532)	0.849	1.122 (0.272–4.638)	0.873	0.915 (0.226–3.702)	0.900	1.248 (0.301–5.173)	0.760
Not specified	1.516 (1.171–1.962)	0.002	1.213 (0.898–1.639)	0.208	1.441 (1.103–1.883)	0.007	1.151 (0.843–1.571)	0.368
N								
No	1 [reference]	–	1 [reference]	–	1 [reference]	–	1 [reference]	–
N1	1.223 (0.924–1.617)	0.159	1.203 (0.904–1.601)	0.205	1.267 (0.950–1.690)	0.108	1.243 (0.926–1.669)	0.148
N2	0.971 (0.507–1.861)	0.930	1.041 (0.529–2.047)	0.908	1.051 (0.547–2.020)	0.880	1.144 (0.579–2.260)	0.699
N3	1.354 (0.860–2.130)	0.190	1.392 (0.877–2.208)	0.160	1.398 (0.878–2.225)	0.158	1.424 (0.886–2.287)	0.144
Unknown	1.518 (1.148–2.008)	0.003	1.372 (1.026–1.834)	0.033	1.506 (1.126–2.016)	0.006	1.390 (1.027–1.881)	0.033
T stage								
T1	1 [reference]	–			1 [reference]	–		
T2	1.468 (0.777–2.771)	0.237			1.517 (0.801–2.873)	0.201		
T3	0.923 (0.594–1.436)	0.723			0.909 (0.577–1.430)	0.679		
T4	1.5 (0.968–2.323)	0.069			1.380 (0.874–2.179)	0.167		
Others	1.227 (0.892–1.687)	0.209			1.198 (0.864–1.660)	0.279		
Tumor size								
≤ 5 cm	1 (reference)	–			1 (reference)	–		
> 5 cm	0.993 (0.671–1.470)	0.973			0.982 (0.654–1.473)	0.982		
Others	1.148 (0.878–1.500)	0.314			1.145 (0.869–1.510)	0.336		

All significant factors from the univariable analysis were entered into the multivariable Cox regression model. Patients who underwent surgery of primary tumor had significantly better OS compared to patients who did not undergo surgery (adjusted hazard ratio [aHR] 0.452, 95% CI 0.269–0.758). Grade IV patients had a worse OS compared to patients with Grade I tumors (aHR 5.322, 95% CI 1.114–25.415). Patients with primary tumors of antrum (aHR 1.957, 95% CI 1.122–3.414) and greater curvature (aHR 2.339, 95% CI 1.238–4.422) had significantly worse OS compared to patients with primary tumor of cardia. Patients with unknown N stage had significantly worse OS compared to patients at N1 stage (aOR 1.372, 95% CI 1.026–1.834) (Table 3).

Univariable Cox regression analysis of patients with brain metastasis showed that bone metastasis, no surgery, grade IV tumor, unknown N stage and primary tumor of antrum, body, greater curvature and overlapping/non-specified site of primary tumor were significant predictors of worse CSS (Table 3).

All significant factors from the univariable analysis were entered into the multivariable Cox regression model. Patients who underwent surgery for primary tumor had significantly better CSS compared to patients who did not undergo surgery (aHR 0.458, 95% CI 0.268–0.781). Grade IV patients had a worse CSS compared to patients with Grade I tumors (aHR 5.287, 95% CI 1.106–25.268). Patients with primary tumors of antrum (aHR 1.858, 95% CI 1.045–3.302) had significantly worse CSS compared to patients with primary tumor of cardia. Patients with unknown N stage had significantly worse CSS compared to patients at N1 stage (aOR 1.390, 95% CI 1.027–1.881) (Table 3).

## Discussion

Due to the known rarity of brain metastasis in gastric carcinoma patients, the risk factors and prognostic variables have yet to be clearly determined. Our results indicated a greater risk of brain metastasis in patients aged less than 60 years, those who had already developed lung and bone metastasis, patients with grade II, III, and IV cancer, and patients with N3 stage. In contrast, patients who underwent surgery for primary tumor and those with tumors involving antrum and body of stomach had a decreased risk of brain metastasis compared to patients who did not undergo surgery and those with tumors involving cardia of stomach, respectively. Surgery of primary tumor was the best diagnostic predictor for brain metastasis. The median OS and the median CSS were lower in patients with brain metastasis than in those without brain metastasis. Better OS and CSS were seen in patients who underwent surgery for primary tumor; whereas, OS and CSS were worse for patients with primary tumor of antrum and grade IV tumor.

Data from 59,231 patients with gastric carcinoma were included in our current study, and metastasis to the brain was reported in 0.62% of the patients. Our results are comparable to Qui et al. who analysed data of gastric carcinoma patients between the years 2010 and 2014 and reported 0.79% of their study population to have brain metastasis^16^. A study conducted by Lin et al. reported 0.39% of their study population of 18,752 gastric carcinoma patients to have brain metastasis^12^. The variations in the occurrence of brain metastasis can be due to multiple factors, including but not limited to, differences in the study population, treatment of choice, and the duration of treatment.

Existing literature shows gastric carcinoma to have a higher incidence of metastasising to the liver (16.82%), lungs (5.92%), and bone (5.08%)^16^. The observed distribution is in accordance with the path of spread of tumor cells, with metastasis being most commonly reported in the site closest to the stomach. Tumor cells from the stomach reach the liver through the portal veins, before spreading to the lungs and finally to the brain. The documented occurrence of brain metastasis could potentially be underestimated, as routine brain scans are not typically conducted during the evaluation of gastric cancer cases^17^. Moreover, owing to rapid disease progression and shorter survival time in many patients with brain metastasis, there is a lack of clinical information^12^. Thus, it may be safe to assume that the incidence of brain metastasis in gastric carcinoma patients is underestimated.

Other gastrointestinal cancers, especially colorectal and oesophageal are known to be more widely associated with brain metastasis, in comparison to gastric and pancreatic cancers. This difference may be due to different genetic makeups, as well as diverging mechanisms and routes of haematological dissemination^6^.

The risk of brain metastasis was lesser in patients with primary tumor of antrum but they had significantly worse OS compared to primary proximal tumors. Our findings are consistent with Qui et al. who report that metastasis was significantly more likely to occur in proximal stomach cancer compared to distal stomach cancer. Yang et al. also reported a survival benefit in tumors of gastric cardia compared to more distal locations^13,16^. However, according to another study, proximal tumors were reported to be larger, with deeper penetration of gastric wall, more frequent metastasis to lymph nodes and more advanced stage. Overall survival was worse in proximal gastric tumors^18^. The disparity in available literature warrants more extensive research into this matter.

As expected, patients with grade IV tumors had a worse OS and CSS compared to those with grade I tumors. This was consistent with the findings of Yang et al.^13^. Studies conducted on bone metastasis in gastric cancer patients also report a similar pattern of prognosis and overall survival^3^. Our study suggested a higher occurrence of brain metastasis in younger patients which may be attributed to the differences in lymph node involvement in different age groups. The proportion of gastric cancer patients with more than 15 lymph node metastases decreases significantly with age^19^. It should also be highlighted here that younger age was not a significant predictor of OS and CSS. A study on gastric carcinoma in young patients also reported similar results^20^.

In our study, the mean overall survival in patients with brain metastasis was 3 months, as opposed to 17 months in patients without brain involvement. This was consistent with the findings reported by Qui et al.^16^. Mean cancer survival was also significantly lesser in patients with brain metastasis (3 months versus 22 months). A lower survival rate correlates with the fact that brain metastasis from gastric cancer is a late event in the disease’s clinical course. Moreover, the existence of the blood–brain barrier hinders the maximal therapeutic effect of chemotherapeutic drugs, resulting in a poor prognosis^21^.

Among the various risk factors assessed in our analysis, surgery was seen to be the best diagnostic predictor for brain metastasis. Patients who underwent surgery had a significantly lower risk of developing brain metastasis and had a better OS compared to those who did not undergo surgery. However, once brain metastasis has occurred, various prognostic factors like performance status, number and site of metastases and dissemination to other organs, need to be considered before forming a treatment plan. It is known the aggressive nature of brain metastasis in gastric carcinoma warrants an aggressive treatment like neurosurgery, combined with stereotactic radiosurgery, palliative radiotherapy, and chemotherapy^11^.

This is the first SEER-based study to focus solely on brain metastasis in gastric cancer patients. Limitations of our analysis should be considered while interpreting the results. Despite a large study population, selection bias exists owing to the retrospective study design. Other important prognostic factors, like imaging, CEA and alkaline phosphatase levels, and various treatment regimens have not been studied. Data from SEER included reported cases of gastric carcinoma from 2010 to 2019; it may be argued that this provides an inadequate follow-up period. Furthermore, ethnicity and geographical factors, which are known risk factors for gastric cancer, have been not extensively studied.

In conclusion, the incidence of brain metastasis for gastric cancer patients is reported to be 0.62%. The presence of brain metastasis significantly reduces the overall and cancer-specific survival, and higher staging and grading are associated with a worse prognosis. Clinicians need to consider clinicopathological factors when deciding on diagnostic and treatment regimens. Further research, including other prognostic factors such as the number and site of brain metastases, various treatment options and a larger prospective cohort, is imperative.

## Data Availability

The datasets used and analysed during the current study available from the corresponding author on reasonable request.

## References

[1] Sung H, Ferlay J, Siegel RL, Laversanne M, Soerjomataram I, Jemal A (2021). Global cancer statistics 2020: GLOBOCAN estimates of incidence and mortality worldwide for 36 cancers in 185 countries. CA Cancer J. Clin..

[2] Gao JP, Xu W, Liu WT, Yan M, Zhu ZG (2018). Tumor heterogeneity of gastric cancer: From the perspective of tumor-initiating cell. World J. Gastroenterol..

[3] Xiaobin C, Zhaojun X, Tao L, Tianzeng D, Xuemei H, Fan Z (2022). Analysis of related risk factors and prognostic factors of gastric cancer with bone metastasis: A SEER-based study. J. Immunol. Res..

[4] Brenner H, Rothenbacher D, Arndt V (2009). Epidemiology of stomach cancer. Methods Mol Biol..

[5] Rawla P, Barsouk A (2019). Epidemiology of gastric cancer: Global trends, risk factors and prevention. Prz. Gastroenterol..

[6] Lemke J, Scheele J, Kapapa T, von Karstedt S, Wirtz CR, Henne-Bruns D (2014). Brain metastases in gastrointestinal cancers: Is there a role for surgery?. Int. J. Mol. Sci..

[7] Barnholtz-Sloan JS, Sloan AE, Davis FG, Vigneau FD, Lai P, Sawaya RE (2004). Incidence proportions of brain metastases in patients diagnosed (1973 to 2001) in the Metropolitan Detroit Cancer Surveillance System. J. Clin. Oncol..

[8] Riihimäki M, Hemminki A, Sundquist K, Sundquist J, Hemminki K (2016). Metastatic spread in patients with gastric cancer. Oncotarget..

[9] York JE, Stringer J, Ajani JA, Wildrick DM, Gokaslan ZL (1999). Gastric cancer and metastasis to the brain. Ann. Surg. Oncol..

[10] Kasakura Y, Fujii M, Mochizuki F, Suzuki T, Takahashi T (2000). Clinicopathological study of brain metastasis in gastric cancer patients. Surg Today..

[11] Kraszkiewicz M, Wydmanski J (2014). Brain metastases from stomach cancer: The role of different treatment modalities and efficacy of palliative radiotherapy. Rep. Pract. Oncol. Radiother..

[12] Lin Z, Wang R, Zhou Y, Wang Q, Yang C-Y, Hao B-C (2022). Prediction of distant metastasis and survival prediction of gastric cancer patients with metastasis to the liver, lung, bone, and brain: Research based on the SEER database. Ann. Transl. Med..

[13] Yang D, Hendifar A, Lenz C, Togawa K, Lenz F, Lurje G (2011). Survival of metastatic gastric cancer: Significance of age, sex and race/ethnicity. J. Gastrointest. Oncol..

[14] *Surveillance, Epidemiology, and End Results (SEER) Program* (www.seer.cancer.gov) SEER*Stat Database: Incidence—SEER Research Data, 17 Registries, Nov 2021 Sub (2000–2019)—Linked To County Attributes—Time Dependent (1990–2019) Income/Rurality, 1969–2.

[15] *Surveillance Research Program*. National Cancer Institute SEER*Stat software (www.seer.cancer.gov/seerstat) Version 8.4.0.1.

[16] Qiu MZ, Shi SM, Chen ZH, Yu HE, Sheng H, Jin Y (2018). Frequency and clinicopathological features of metastasis to liver, lung, bone, and brain from gastric cancer: A SEER-based study. Cancer Med..

[17] Lohr F, Pirzkall A, Hof H, Fleckenstein K, Debus J (2001). Adjuvant treatment of brain metastases. Semin. Surg. Oncol..

[18] Pinto-de-Sousa J, David L, Seixas M, Pimenta A (2001). Clinicopathologic profiles and prognosis of gastric carcinomas from the cardia, fundus/body and antrum. Dig. Surg..

[19] Ahmad A, Khan H, Cholankeril G, Katz SC, Somasundar P (2016). The impact of age on nodal metastases and survival in gastric cancer. J. Surg. Res..

[20] Al-Refaie WB, Pisters PW, Chang GJ (2007). Gastric adenocarcinoma in young patients: A population-based appraisal. Ann. Surg. Oncol..

[21] Daneman R, Prat A (2015). The blood–brain barrier. Cold Spring Harb. Perspect. Biol..

